# 4-Hy­droxy-2-methyl-1,1-dioxo-2*H*-1λ^6^,2- benzothia­zine-3-carb­oxy­lic acid hemihydrate

**DOI:** 10.1107/S1600536812004291

**Published:** 2012-02-04

**Authors:** Farhana Aman, Waseeq Ahmad Siddiqui, Adnan Ashraf, M. Nawaz Tahir

**Affiliations:** a University of Sargodha, Department of Chemistry, Sargodha, Pakistan; b University of Sargodha, Department of Physics, Sargodha, Pakistan

## Abstract

In the title compound, C_10_H_9_NO_5_S·0.5H_2_O, two geometrically different organic mol­ecules are present. The benzene rings and the carboxyl­ate groups are oriented at dihedral angles of 13.44 (4) and 21.15 (18)°. In both mol­ecules, an intra­molecular O—H⋯O hydrogen bond generates an *S*(6) ring. In the crystal, both moleucles form inversion dimers linked by pairs of O—H⋯O hydrogen bonds to generate *R*_2_^2^(8) loops. The dimers are consolidated into chains extending along [100] by bridging O—H⋯O hydrogen bonds from the water mol­ecule. A weak C—H⋯O hydrogen bond also occurs.

## Related literature

For background to non-steroidal anti-inflammatory drugs, see: Akram *et al.* (2008 ▶); Foye *et al.* (1995 ▶); Lombardino *et al.* (1971 ▶, 1973 ▶); Siddiqui, Ahmad, Siddiqui *et al.* (2008 ▶); Siddiqui, Ahmad, Tariq *et al.* (2008 ▶). For a related structure, see: Golič & Leban (1987 ▶). For graph-set notation, see: Bernstein *et al.* (1995 ▶). For puckering parameters, see: Cremer & Pople (1975 ▶).
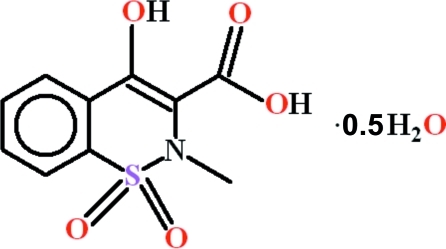


## Experimental

### 

#### Crystal data

C_10_H_9_NO_5_S·0.5H_2_O
*M* *_r_* = 264.25
Triclinic, 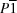

*a* = 7.1837 (2) Å
*b* = 8.5847 (3) Å
*c* = 17.9814 (4) Å
α = 87.605 (1)°
β = 89.713 (2)°
γ = 87.174 (1)°
*V* = 1106.59 (6) Å^3^
*Z* = 4
Mo *K*α radiation
μ = 0.31 mm^−1^
*T* = 296 K
0.35 × 0.25 × 0.22 mm

#### Data collection

Bruker Kappa APEXII CCD diffractometer
Absorption correction: multi-scan (*SADABS*; Bruker, 2005 ▶) *T*_min_ = 0.915, *T*_max_ = 0.938
15712 measured reflections
4317 independent reflections
3468 reflections with *I* > 2σ(*I*)
*R*_int_ = 0.028

#### Refinement

*R*[*F* ^2^ > 2σ(*F* ^2^)] = 0.038
*wR*(*F* ^2^) = 0.097
*S* = 1.03
4317 reflections
295 parameters
H atoms treated by a mixture of independent and constrained refinement
Δρ_max_ = 0.32 e Å^−3^
Δρ_min_ = −0.32 e Å^−3^

### 

Data collection: *APEX2* (Bruker, 2009 ▶); cell refinement: *SAINT* (Bruker, 2009 ▶); data reduction: *SAINT*; program(s) used to solve structure: *SHELXS97* (Sheldrick, 2008 ▶); program(s) used to refine structure: *SHELXL97* (Sheldrick, 2008 ▶); molecular graphics: *ORTEP-3 for Windows* (Farrugia, 1997 ▶) and *PLATON* (Spek, 2009 ▶); software used to prepare material for publication: *WinGX* (Farrugia, 1999 ▶) and *PLATON*.

## Supplementary Material

Crystal structure: contains datablock(s) global, I. DOI: 10.1107/S1600536812004291/hb6618sup1.cif

Structure factors: contains datablock(s) I. DOI: 10.1107/S1600536812004291/hb6618Isup2.hkl

Supplementary material file. DOI: 10.1107/S1600536812004291/hb6618Isup3.cml

Additional supplementary materials:  crystallographic information; 3D view; checkCIF report

## Figures and Tables

**Table 1:** Hydrogen-bond geometry (Å, °)

*D*—H⋯*A*	*D*—H	H⋯*A*	*D*⋯*A*	*D*—H⋯*A*
O1—H1⋯O2^i^	0.83 (2)	1.82 (2)	2.647 (2)	177 (2)
O3—H3⋯O2	0.88 (2)	1.76 (2)	2.558 (2)	150 (2)
O6—H6*A*⋯O11^ii^	0.89	1.70	2.5881 (17)	170
O8—H8*A*⋯O7	0.88	1.75	2.5675 (8)	154
O11—H11*A*⋯O4^iii^	0.81 (3)	2.28 (3)	3.029 (2)	155 (3)
O11—H11*B*⋯O7^iii^	0.84 (3)	2.06 (3)	2.8508 (18)	158 (3)
C10—H10*B*⋯O1^iv^	0.96	2.50	3.3679 (16)	151

Symmetry codes: (i) 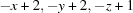; (ii) 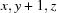; (iii) 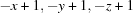; (iv) 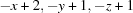.

**Fig. 1.**
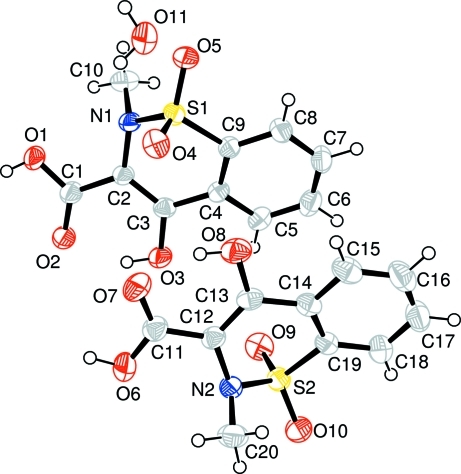
View of the title compound with displacement ellipsoids drawn at the 50% probability level.

**Fig. 2.**
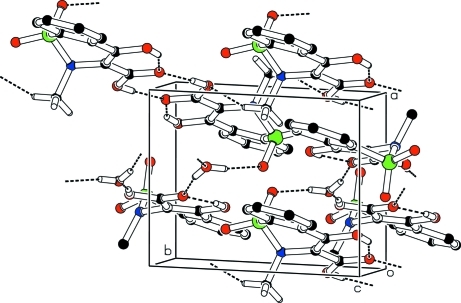
The partial packing, which shows that molecules form polymeric chains extending along the [100] direction.

## supplementary crystallographic information

### Comment

Among non-steroidal anti-inflammatory drugs (NSAIDs) some of the agents who have proven clinical efficacy are the β-diketones such as phenylbutazone or derivatives of the carboxylic acid class like aspirin, indomethacin and flufenamic acid, (Lombardino *et al.*, 1971). Piroxicam is the first member of the 'Oxicam' class of NSAIDs to serve as an effective anti-inflammatory agent and gain immediate popularity in the international market (Foye *et al.*, 1995). The carboxylic acid derivative of piroxicam, 4-hydroxy-2-methyl-2*H*-1,2 benzothiazine-3-carboxylic acid 1,1-dioxide has shown satisfactory plasma half-life (Lombardino *et al.*, 1973) for its application in human beings. Due to our interest in the synthesis of this type of molecules and their transition metal complexes (Siddiqui, Ahmad, Siddiqui *et al.*, 2008; Siddiqui, Ahmad, Tariq *et al.*, 2008; Akram *et al.*, 2008), we have synthesized this molecule by a simple procedure. Herein, we report the crystal structure of (I), (Fig. 1).

The crystal structures of the reactant i.e (II) methyl 4-hydroxy-2-methyl-2*H*-1,2-benzothiazine-3-carboxylate-1,1-dioxide (Golič & Leban, 1987) has been published which is also related to (I).

The asymmetric unit of (I) consists of two molecules (A & B) of the acid moiety and a water molecule. In the molecule A, the benzene ring C (C4–C9) is planar with r. m. s. deviation of 0.0019 Å and oriented at a dihedral angle of 21.15 (18)° with the carboxylate group D (O1/C1/O2). The puckering parameters (Cremer & Pople, 1975) of the adjacent heterocyclic ring E (C2/C3/C4/C9/S1/N1) are Q = 0.5183 (15) Å, θ = 114.4 (2)° and φ = 204.0 (2)°. The molecule B differs from A as the benzene ring F (C14—C1) [r. m. s. deviation = 0.0063 Å] is oriented at a dihedral angle of 13.44 (4)° with its carboxylate moiety G (O6/C11/O7). Also the puckering parameters of its adjacent heterocyclic ring H (C12/C13/C14/C19/S2/N2) are Q = 0.5047 (7) Å, θ = 117.06 (10)° and φ = 205.52 (13)°. In both molecules there exist intramolecular H-bonding of O—H···O type (Table 1, Fig. 2) forming S(6) ring motif (Bernstein *et al.*, 1995). The molecules are dimerized due to intermolecular H-bonding of O—H···O bonding (Table 1, Fig. 2) forming a conventional *R*_2_^2^(8) ring motifs. The water molecules interlink the dimers from the CO_2_ and SO_2_ groups due to O—H···O bonding, where CO_2_ and SO_2_ are of molecule B and A, respectively. The molecules are stabilized in the form of one-dimensional polymeric chains along the [1 0 0] direction.

### Experimental

A light yellow solution of 4-hydroxy-2-methyl-2*H*-1,2 benzothiazine-3-carboxylic acid 1,1 dioxide methyl ester (0.5 g, 1.86 mmol) was subjected to stirring with an excess of sodium hydroxide at room temperature for 24 h. The reaction mixture was then acidified with dilute HCl and the precipitates filtered, washed with cold distilled water (3×25 ml) and dried to get white product (0.4 g, 1.65 mmol, 89%). m.p 361–362 K.

### Refinement

The coordinates of hydroxy H-atoms were refined. The H-atoms of aryl and methyl groups were positioned geometrically at C—H = 0.93 and C—H = 0.96 Å, respectively and included in the refinement as riding with *U*_iso_(H) = *xU*_eq_(C, O), where *x* = 1.5 for methyl groups and *x* = 1.2 for all other H atoms.

### Crystal data

**Table:**

C_10_H_9_NO_5_S·0.5H_2_O	*Z* = 4
*M**_r_* = 264.25	*F*(000) = 548
Triclinic, *P*1	*D*_x_ = 1.586 Mg m^−^^3^
Hall symbol: -P 1	Mo *K*α radiation, λ = 0.71073 Å
*a* = 7.1837 (2) Å	Cell parameters from 3468 reflections
*b* = 8.5847 (3) Å	θ = 2.3–26.0°
*c* = 17.9814 (4) Å	µ = 0.31 mm^−^^1^
α = 87.605 (1)°	*T* = 296 K
β = 89.713 (2)°	Prism, light green
γ = 87.174 (1)°	0.35 × 0.25 × 0.22 mm
*V* = 1106.59 (6) Å^3^	

### Data collection

**Table:**

Bruker Kappa APEXII CCD diffractometer	4317 independent reflections
Radiation source: fine-focus sealed tube	3468 reflections with *I* > 2σ(*I*)
Graphite monochromator	*R*_int_ = 0.028
Detector resolution: 8.00 pixels mm^-1^	θ_max_ = 26.0°, θ_min_ = 2.3°
ω scans	*h* = −7→8
Absorption correction: multi-scan (*SADABS*; Bruker, 2005)	*k* = −9→10
*T*_min_ = 0.915, *T*_max_ = 0.938	*l* = −22→22
15712 measured reflections	

### Refinement

**Table:**

Refinement on *F*^2^	Secondary atom site location: difference Fourier map
Least-squares matrix: full	Hydrogen site location: inferred from neighbouring sites
*R*[*F*^2^ > 2σ(*F*^2^)] = 0.038	H atoms treated by a mixture of independent and constrained refinement
*wR*(*F*^2^) = 0.097	*w* = 1/[σ^2^(*F*_o_^2^) + (0.0439*P*)^2^ + 0.3985*P*] where *P* = (*F*_o_^2^ + 2*F*_c_^2^)/3
*S* = 1.03	(Δ/σ)_max_ < 0.001
4317 reflections	Δρ_max_ = 0.32 e Å^−^^3^
295 parameters	Δρ_min_ = −0.32 e Å^−^^3^
0 restraints	Extinction correction: *SHELXL97* (Sheldrick, 2008), Fc^*^=kFc[1+0.001xFc^2^λ^3^/sin(2θ)]^-1/4^
Primary atom site location: structure-invariant direct methods	Extinction coefficient: 0.0115 (13)

### Special details

**Table:**

Geometry. Bond distances, angles *etc*. have been calculated using the rounded fractional coordinates. All su's are estimated from the variances of the (full) variance-covariance matrix. The cell e.s.d.'s are taken into account in the estimation of distances, angles and torsion angles
Refinement. Refinement of *F*^2^ against ALL reflections. The weighted *R*-factor *wR* and goodness of fit *S* are based on *F*^2^, conventional *R*-factors *R* are based on *F*, with *F* set to zero for negative *F*^2^. The threshold expression of *F*^2^ > σ(*F*^2^) is used only for calculating *R*-factors(gt) *etc*. and is not relevant to the choice of reflections for refinement. *R*-factors based on *F*^2^ are statistically about twice as large as those based on *F*, and *R*- factors based on ALL data will be even larger.

### Fractional atomic coordinates and isotropic or equivalent isotropic displacement parameters (Å^2^)

**Table:**

	*x*	*y*	*z*	*U*_iso_*/*U*_eq_	
S1	0.73268 (7)	0.47997 (5)	0.36202 (3)	0.0326 (2)	
O1	1.0126 (2)	0.78588 (17)	0.48912 (8)	0.0460 (5)	
O2	0.9190 (2)	0.99420 (16)	0.41728 (8)	0.0487 (5)	
O3	0.7800 (2)	0.95223 (16)	0.28936 (8)	0.0429 (5)	
O4	0.56659 (19)	0.53462 (17)	0.39886 (8)	0.0434 (5)	
O5	0.7877 (2)	0.31809 (15)	0.36788 (8)	0.0433 (5)	
N1	0.9045 (2)	0.57982 (17)	0.39058 (8)	0.0318 (5)	
C1	0.9390 (3)	0.8508 (2)	0.42875 (11)	0.0374 (7)	
C2	0.8833 (3)	0.7439 (2)	0.37332 (10)	0.0318 (6)	
C3	0.8093 (3)	0.7998 (2)	0.30725 (10)	0.0318 (6)	
C4	0.7527 (3)	0.6968 (2)	0.24926 (10)	0.0318 (6)	
C5	0.7316 (3)	0.7497 (3)	0.17540 (11)	0.0396 (7)	
C6	0.6781 (3)	0.6499 (3)	0.12229 (11)	0.0458 (8)	
C7	0.6430 (3)	0.4970 (3)	0.14134 (12)	0.0462 (8)	
C8	0.6621 (3)	0.4403 (3)	0.21428 (11)	0.0407 (7)	
C9	0.7164 (3)	0.5410 (2)	0.26757 (10)	0.0328 (6)	
C10	1.09513 (6)	0.50694 (9)	0.39024 (3)	0.0442 (7)	
S2	0.34876 (5)	1.12692 (4)	0.12496 (2)	0.0377 (2)	
O6	0.36746 (6)	1.17536 (7)	0.35142 (3)	0.0544 (6)	
O7	0.37431 (6)	0.91858 (7)	0.37872 (3)	0.0550 (6)	
O8	0.31763 (5)	0.73224 (5)	0.27403 (4)	0.0476 (5)	
O9	0.54647 (5)	1.10602 (4)	0.13153 (2)	0.0479 (5)	
O10	0.27227 (5)	1.25250 (4)	0.07867 (2)	0.0530 (6)	
N2	0.26231 (5)	1.14344 (4)	0.20849 (2)	0.0361 (5)	
C11	0.35007 (5)	1.03047 (5)	0.33412 (3)	0.0412 (7)	
C12	0.29810 (5)	1.00779 (4)	0.25703 (2)	0.0352 (6)	
C13	0.28734 (5)	0.86196 (5)	0.23138 (3)	0.0352 (6)	
C14	0.24568 (5)	0.83233 (5)	0.15354 (3)	0.0363 (6)	
C15	0.19471 (5)	0.68585 (5)	0.13242 (4)	0.0488 (8)	
C16	0.1577 (3)	0.6612 (3)	0.05880 (15)	0.0585 (9)	
C17	0.1707 (3)	0.7789 (3)	0.00546 (14)	0.0598 (9)	
C18	0.2239 (3)	0.9247 (3)	0.02421 (12)	0.0511 (8)	
C19	0.2620 (3)	0.9500 (2)	0.09812 (11)	0.0372 (6)	
C20	0.0766 (3)	1.2219 (3)	0.21567 (13)	0.0530 (8)	
O11	0.4465 (3)	0.2097 (2)	0.48986 (9)	0.0635 (7)	
H1	1.038 (3)	0.854 (3)	0.5179 (13)	0.0552*	
H3	0.816 (3)	1.001 (3)	0.3286 (13)	0.0514*	
H5	0.75359	0.85293	0.16185	0.0475*	
H6	0.66556	0.68614	0.07302	0.0549*	
H7	0.60624	0.43125	0.10490	0.0554*	
H8	0.63897	0.33706	0.22725	0.0488*	
H10A	1.14009	0.50333	0.33994	0.0663*	
H10B	1.09340	0.40282	0.41180	0.0663*	
H10C	1.17576	0.56731	0.41874	0.0663*	
H6A	0.39669	1.17567	0.39964	0.0652*	
H8A	0.33841	0.76910	0.31793	0.0571*	
H15	0.18570	0.60481	0.16802	0.0585*	
H16	0.12344	0.56331	0.04515	0.0702*	
H17	0.14353	0.76055	−0.04383	0.0717*	
H18	0.23387	1.00439	−0.01205	0.0613*	
H20A	0.05271	1.24259	0.26698	0.0795*	
H20B	−0.01620	1.15579	0.19802	0.0795*	
H20C	0.07219	1.31847	0.18667	0.0795*	
H11A	0.453 (4)	0.294 (4)	0.5075 (17)	0.0762*	
H11B	0.507 (4)	0.152 (3)	0.5209 (16)	0.0762*	

### Atomic displacement parameters (Å^2^)

**Table:**

	*U*^11^	*U*^22^	*U*^33^	*U*^12^	*U*^13^	*U*^23^
S1	0.0377 (3)	0.0276 (3)	0.0328 (3)	−0.0056 (2)	−0.0022 (2)	−0.0010 (2)
O1	0.0701 (10)	0.0310 (8)	0.0377 (8)	−0.0098 (7)	−0.0214 (7)	0.0008 (6)
O2	0.0744 (11)	0.0265 (8)	0.0457 (9)	−0.0063 (7)	−0.0213 (8)	−0.0001 (6)
O3	0.0589 (10)	0.0283 (8)	0.0412 (8)	−0.0037 (7)	−0.0138 (7)	0.0047 (6)
O4	0.0401 (8)	0.0479 (9)	0.0427 (8)	−0.0068 (7)	0.0066 (6)	−0.0051 (7)
O5	0.0575 (9)	0.0267 (8)	0.0456 (8)	−0.0047 (7)	−0.0087 (7)	0.0023 (6)
N1	0.0350 (9)	0.0247 (8)	0.0356 (9)	−0.0021 (7)	−0.0076 (7)	0.0014 (7)
C1	0.0440 (12)	0.0310 (12)	0.0374 (11)	−0.0070 (9)	−0.0078 (9)	0.0029 (9)
C2	0.0360 (10)	0.0255 (10)	0.0338 (10)	−0.0034 (8)	−0.0046 (8)	0.0020 (8)
C3	0.0327 (10)	0.0270 (10)	0.0357 (10)	−0.0028 (8)	−0.0009 (8)	0.0014 (8)
C4	0.0286 (10)	0.0338 (11)	0.0326 (10)	0.0005 (8)	−0.0021 (8)	0.0008 (8)
C5	0.0402 (11)	0.0423 (12)	0.0357 (11)	−0.0018 (9)	−0.0028 (9)	0.0048 (9)
C6	0.0468 (13)	0.0589 (15)	0.0311 (11)	0.0020 (11)	−0.0041 (9)	−0.0003 (10)
C7	0.0489 (13)	0.0524 (14)	0.0380 (12)	0.0010 (11)	−0.0087 (10)	−0.0138 (10)
C8	0.0424 (12)	0.0368 (12)	0.0435 (12)	−0.0008 (9)	−0.0056 (9)	−0.0090 (9)
C9	0.0308 (10)	0.0341 (11)	0.0334 (10)	0.0008 (8)	−0.0030 (8)	−0.0026 (8)
C10	0.0401 (12)	0.0345 (12)	0.0571 (14)	0.0004 (9)	−0.0016 (10)	0.0079 (10)
S2	0.0490 (3)	0.0330 (3)	0.0308 (3)	−0.0030 (2)	0.0016 (2)	0.0025 (2)
O6	0.0779 (12)	0.0512 (10)	0.0340 (8)	−0.0002 (8)	−0.0103 (8)	−0.0042 (7)
O7	0.0713 (11)	0.0566 (10)	0.0354 (8)	0.0034 (9)	−0.0033 (7)	0.0105 (8)
O8	0.0563 (10)	0.0377 (9)	0.0475 (9)	−0.0017 (7)	0.0050 (7)	0.0112 (7)
O9	0.0454 (9)	0.0476 (9)	0.0509 (9)	−0.0086 (7)	0.0081 (7)	0.0020 (7)
O10	0.0814 (12)	0.0390 (9)	0.0373 (8)	0.0008 (8)	−0.0052 (8)	0.0091 (7)
N2	0.0467 (10)	0.0312 (9)	0.0297 (9)	0.0020 (8)	0.0000 (7)	0.0009 (7)
C11	0.0430 (12)	0.0462 (13)	0.0336 (11)	0.0028 (10)	0.0028 (9)	0.0029 (10)
C12	0.0382 (11)	0.0363 (12)	0.0304 (10)	0.0015 (9)	0.0023 (8)	0.0038 (9)
C13	0.0328 (10)	0.0334 (11)	0.0384 (11)	0.0001 (9)	0.0048 (8)	0.0076 (9)
C14	0.0304 (10)	0.0345 (11)	0.0439 (11)	−0.0009 (9)	0.0043 (8)	−0.0032 (9)
C15	0.0429 (12)	0.0387 (13)	0.0653 (15)	−0.0065 (10)	0.0060 (11)	−0.0055 (11)
C16	0.0538 (15)	0.0506 (15)	0.0735 (18)	−0.0075 (12)	−0.0037 (12)	−0.0250 (14)
C17	0.0626 (16)	0.0646 (17)	0.0533 (15)	0.0035 (13)	−0.0080 (12)	−0.0242 (14)
C18	0.0618 (15)	0.0530 (15)	0.0384 (12)	0.0022 (12)	−0.0019 (10)	−0.0082 (11)
C19	0.0393 (11)	0.0346 (11)	0.0376 (11)	0.0021 (9)	0.0008 (9)	−0.0039 (9)
C20	0.0598 (15)	0.0465 (14)	0.0504 (14)	0.0160 (12)	0.0057 (11)	0.0017 (11)
O11	0.0951 (14)	0.0557 (12)	0.0398 (9)	0.0004 (11)	−0.0156 (9)	−0.0064 (8)

### Geometric parameters (Å, º)

**Table:**

S1—O4	1.4302 (15)	C4—C9	1.401 (2)
S1—O5	1.4257 (14)	C5—C6	1.378 (3)
S1—N1	1.6339 (15)	C6—C7	1.378 (4)
S1—C9	1.7590 (19)	C7—C8	1.385 (3)
S2—N2	1.6314 (5)	C8—C9	1.387 (3)
S2—O9	1.4276 (5)	C5—H5	0.9300
S2—O10	1.4241 (5)	C6—H6	0.9300
S2—C19	1.7566 (18)	C7—H7	0.9300
O1—C1	1.302 (2)	C8—H8	0.9300
O2—C1	1.241 (2)	C10—H10C	0.9600
O3—C3	1.341 (2)	C10—H10A	0.9600
O1—H1	0.83 (2)	C10—H10B	0.9600
O3—H3	0.88 (2)	C11—C12	1.4607 (6)
O6—C11	1.3067 (7)	C12—C13	1.3580 (6)
O7—C11	1.2315 (7)	C13—C14	1.4679 (7)
O8—C13	1.3349 (7)	C14—C19	1.3977 (19)
O6—H6A	0.8900	C14—C15	1.3951 (6)
O8—H8A	0.8800	C15—C16	1.379 (3)
O11—H11B	0.84 (3)	C16—C17	1.370 (4)
O11—H11A	0.81 (3)	C17—C18	1.382 (4)
N1—C10	1.4774 (15)	C18—C19	1.386 (3)
N1—C2	1.431 (2)	C15—H15	0.9300
N2—C12	1.4394 (5)	C16—H16	0.9300
N2—C20	1.472 (2)	C17—H17	0.9300
C1—C2	1.453 (3)	C18—H18	0.9300
C2—C3	1.363 (3)	C20—H20C	0.9600
C3—C4	1.467 (3)	C20—H20A	0.9600
C4—C5	1.392 (3)	C20—H20B	0.9600
			
O4—S1—O5	119.32 (9)	C7—C6—H6	120.00
O4—S1—N1	107.88 (8)	C6—C7—H7	120.00
O4—S1—C9	108.12 (9)	C8—C7—H7	120.00
O5—S1—N1	108.56 (8)	C9—C8—H8	121.00
O5—S1—C9	109.51 (9)	C7—C8—H8	121.00
N1—S1—C9	102.07 (9)	N1—C10—H10B	109.00
O9—S2—O10	119.03 (3)	N1—C10—H10C	109.00
O9—S2—N2	107.87 (3)	N1—C10—H10A	109.00
O9—S2—C19	108.08 (7)	H10A—C10—H10B	109.00
O10—S2—N2	108.36 (3)	H10B—C10—H10C	109.00
O10—S2—C19	109.87 (7)	H10A—C10—H10C	109.00
N2—S2—C19	102.34 (7)	O6—C11—O7	123.53 (5)
C1—O1—H1	109.6 (17)	O6—C11—C12	115.45 (4)
C3—O3—H3	105.2 (16)	O7—C11—C12	121.03 (4)
C11—O6—H6A	108.00	N2—C12—C11	118.50 (3)
C13—O8—H8A	103.00	N2—C12—C13	120.77 (4)
H11A—O11—H11B	102 (3)	C11—C12—C13	120.72 (4)
S1—N1—C2	114.15 (12)	C12—C13—C14	123.01 (4)
S1—N1—C10	118.74 (10)	O8—C13—C12	123.29 (5)
C2—N1—C10	117.70 (13)	O8—C13—C14	113.69 (4)
S2—N2—C20	117.81 (9)	C13—C14—C15	121.33 (5)
S2—N2—C12	113.99 (3)	C13—C14—C19	120.41 (8)
C12—N2—C20	115.64 (10)	C15—C14—C19	118.23 (9)
O1—C1—O2	123.45 (18)	C14—C15—C16	120.03 (12)
O1—C1—C2	115.62 (15)	C15—C16—C17	120.9 (2)
O2—C1—C2	120.91 (18)	C16—C17—C18	120.6 (2)
N1—C2—C1	118.39 (16)	C17—C18—C19	118.7 (2)
C1—C2—C3	120.37 (16)	S2—C19—C14	117.15 (13)
N1—C2—C3	121.22 (16)	S2—C19—C18	121.23 (16)
O3—C3—C4	113.89 (16)	C14—C19—C18	121.47 (17)
O3—C3—C2	123.67 (16)	C16—C15—H15	120.00
C2—C3—C4	122.44 (16)	C14—C15—H15	120.00
C3—C4—C5	121.78 (17)	C15—C16—H16	120.00
C5—C4—C9	118.12 (18)	C17—C16—H16	120.00
C3—C4—C9	120.10 (16)	C18—C17—H17	120.00
C4—C5—C6	120.3 (2)	C16—C17—H17	120.00
C5—C6—C7	120.8 (2)	C17—C18—H18	121.00
C6—C7—C8	120.6 (2)	C19—C18—H18	121.00
C7—C8—C9	118.5 (2)	N2—C20—H20B	109.00
C4—C9—C8	121.72 (18)	N2—C20—H20C	109.00
S1—C9—C4	117.15 (14)	N2—C20—H20A	109.00
S1—C9—C8	121.07 (15)	H20A—C20—H20C	109.00
C6—C5—H5	120.00	H20B—C20—H20C	109.00
C4—C5—H5	120.00	H20A—C20—H20B	109.00
C5—C6—H6	120.00		
			
O4—S1—N1—C2	−61.72 (14)	C1—C2—C3—C4	179.38 (19)
O4—S1—N1—C10	152.50 (10)	O3—C3—C4—C9	−158.93 (19)
O5—S1—N1—C2	167.65 (13)	C2—C3—C4—C5	−160.1 (2)
O5—S1—N1—C10	21.87 (13)	C2—C3—C4—C9	20.5 (3)
C9—S1—N1—C2	52.05 (14)	O3—C3—C4—C5	20.4 (3)
C9—S1—N1—C10	−93.72 (12)	C3—C4—C5—C6	−179.9 (2)
O4—S1—C9—C4	78.31 (18)	C3—C4—C9—S1	2.5 (3)
O4—S1—C9—C8	−99.01 (19)	C3—C4—C9—C8	179.8 (2)
O5—S1—C9—C4	−150.19 (16)	C9—C4—C5—C6	−0.6 (3)
O5—S1—C9—C8	32.5 (2)	C5—C4—C9—C8	0.4 (3)
N1—S1—C9—C4	−35.29 (18)	C5—C4—C9—S1	−176.86 (16)
N1—S1—C9—C8	147.39 (18)	C4—C5—C6—C7	0.6 (3)
C19—S2—N2—C12	52.04 (8)	C5—C6—C7—C8	−0.5 (3)
C19—S2—N2—C20	−88.35 (13)	C6—C7—C8—C9	0.3 (3)
O9—S2—C19—C14	78.38 (14)	C7—C8—C9—S1	176.90 (17)
O9—S2—C19—C18	−97.21 (18)	C7—C8—C9—C4	−0.3 (3)
O10—S2—C19—C14	−150.27 (11)	O6—C11—C12—N2	−2.89 (5)
O10—S2—C19—C18	34.1 (2)	O6—C11—C12—C13	175.85 (4)
N2—S2—C19—C14	−35.32 (15)	O7—C11—C12—N2	176.85 (4)
N2—S2—C19—C18	149.09 (17)	O7—C11—C12—C13	−4.41 (6)
O10—S2—N2—C20	27.70 (12)	N2—C12—C13—O8	−179.45 (4)
O9—S2—N2—C12	−61.81 (4)	N2—C12—C13—C14	2.10 (6)
O9—S2—N2—C20	157.80 (11)	C11—C12—C13—O8	1.84 (6)
O10—S2—N2—C12	168.08 (3)	C11—C12—C13—C14	−176.62 (3)
S1—N1—C2—C1	140.26 (16)	O8—C13—C14—C15	15.95 (5)
S1—N1—C2—C3	−38.4 (2)	O8—C13—C14—C19	−162.03 (11)
C10—N1—C2—C1	−73.6 (2)	C12—C13—C14—C15	−165.46 (4)
C10—N1—C2—C3	107.7 (2)	C12—C13—C14—C19	16.56 (11)
S2—N2—C12—C11	138.41 (3)	C13—C14—C15—C16	−179.53 (11)
C20—N2—C12—C11	−80.31 (11)	C19—C14—C15—C16	−1.51 (15)
C20—N2—C12—C13	100.95 (11)	C13—C14—C19—S2	4.25 (18)
S2—N2—C12—C13	−40.33 (5)	C13—C14—C19—C18	179.83 (15)
O1—C1—C2—N1	3.0 (3)	C15—C14—C19—S2	−173.79 (8)
O1—C1—C2—C3	−178.26 (19)	C15—C14—C19—C18	1.8 (2)
O2—C1—C2—N1	−178.13 (18)	C14—C15—C16—C17	0.2 (2)
O2—C1—C2—C3	0.6 (3)	C15—C16—C17—C18	0.9 (3)
N1—C2—C3—O3	177.46 (18)	C16—C17—C18—C19	−0.7 (3)
N1—C2—C3—C4	−2.0 (3)	C17—C18—C19—S2	174.68 (17)
C1—C2—C3—O3	−1.2 (3)	C17—C18—C19—C14	−0.7 (3)

### Hydrogen-bond geometry (Å, º)

**Table:**

*D*—H···*A*	*D*—H	H···*A*	*D*···*A*	*D*—H···*A*
O1—H1···O2^i^	0.83 (2)	1.82 (2)	2.647 (2)	177 (2)
O3—H3···O2	0.88 (2)	1.76 (2)	2.558 (2)	150 (2)
O6—H6*A*···O11^ii^	0.89	1.70	2.5881 (17)	170
O8—H8*A*···O7	0.88	1.75	2.5675 (8)	154
O11—H11*A*···O4^iii^	0.81 (3)	2.28 (3)	3.029 (2)	155 (3)
O11—H11*B*···O7^iii^	0.84 (3)	2.06 (3)	2.8508 (18)	158 (3)
C10—H10*B*···O1^iv^	0.96	2.50	3.3679 (16)	151

Symmetry codes: (i) −*x*+2, −*y*+2, −*z*+1; (ii) *x*, *y*+1, *z*; (iii) −*x*+1, −*y*+1, −*z*+1; (iv) −*x*+2, −*y*+1, −*z*+1.
